# A Positive Feedback Loop Between CXCL16 and the Inflammatory Factors IL-17A and TGF-*β* Promotes Large Artery Atherosclerosis by Activating the STAT3/NF-*κ*B Pathway

**DOI:** 10.1155/cdr/2973633

**Published:** 2025-03-24

**Authors:** Xuechen Cui, Yuan Wang, Xuening Li, Hong Li, Ruihua Yin, Yue Liu, Aijun Ma, Shaonan Yang

**Affiliations:** Department of Neurology, The Affiliated Hospital of Qingdao University, Qingdao, China

**Keywords:** atherosclerosis, cxcl16, inflammatory factor, stroke

## Abstract

CXC chemokine ligand 16 (CXCL16) expression is often observed in studies related to atherosclerosis (AS). However, the process by which CXCL16 promotes AS is still unknown. CXCL16 has the potential to be a therapeutic target for atherosclerotic disease, and we studied whether CXCL16 expression in carotid atherosclerotic plaques is correlated with plaque stability. The results revealed that the expression level of CXCL16 in unstable plaques was greater than that in stable plaques (*p* < 0.05). In an in vitro model, CXCL16 promoted the expression of interleukin-17A (IL-17A) and transforming growth factor-*β* (TGF-*β*) and the release of STAT3/NF-*κ*B pathway-associated proteins by regulating the expression of IL-17A, TGF-*β*, and CXCL16. In conclusion, there is a positive feedback regulatory pathway between inflammatory factors and CXCL16 during the progression of carotid AS. Inflammatory factors and CXCL16 promote each other's expression and activate the STAT3/NF-*κ*B pathway to promote carotid AS. CXCL16 is highly expressed in carotid atherosclerotic plaques, affecting plaque stability and further leading to the development of AS-related diseases such as ischaemic stroke. Thus, we hypothesise that CXCL16 is a potential therapeutic target for treating AS and AS-related diseases.

## 1. Introduction

Atherosclerotic disease is the leading cause of death worldwide [1]. Atherosclerotic diseases include coronary artery disease (CAD), stroke, and peripheral artery disease (PAD) [1–3]. In the past few decades, atherosclerosis has been thought to be caused by lipid deposition in the walls of the large and middle arteries [4]. However, in recent years, increasing evidence has shown that inflammation is related to early atherosclerotic formation, the progression of lesions, and the eventual onset of thrombosis [4, 5].

Inflammatory cells gather in the artery wall and participate in and maintain the local inflammatory response, and activated white blood cells and proper artery cells release fibroblast mediators that stimulate the proliferation and migration of smooth muscle cells [6]. By inhibiting collagen synthesis and the expression of collagenase, inflammatory mediators cause the fibre cap to rupture easily, thus contributing to acute thrombotic complications of atherosclerosis [7, 8].

CXCL16 is a multistructural chemokine that functions as a chemokine, adhesion molecule, and scavenger receptor and is involved in many important processes related to AS [9]. In the early stage of AS development, CXCL16 may act as an adhesion factor to mediate monocyte adhesion to vascular endothelial cells and as a chemokine that attracts inflammatory cells to AS sites and induces cytokine secretion [10–13]. With the progression of AS, CXCL16 can also induce vascular smooth muscle cell proliferation and foam cell formation [10, 14].

IL-17A is produced primarily by T helper cells (TH17 cells) [15, 16] and is also produced by other cells, such as macrophages [16]. The relevant literature suggests that IL-17A is significantly expressed in carotid plaques and that the development of atherosclerosis is reduced by the inhibition of IL-17A [15–17]. TGF-*β* has a pleiotropic effect and plays a key role in cell signalling. Studies have shown that TGF-*β* promotes endothelial cell proliferation, migration, angiogenesis, and atherosclerosis [18, 19]. Previous studies have shown that in the tumour environment, CXCL16 has a recruitment effect on monocytes and can promote the transformation of monocytes into macrophages [20], whereas studies on acute coronary syndrome have shown that CXCL16 has a regulatory effect on the secretion of IL-17A and TGF-*β*, among other factors [21, 22].

Signal transduction mediated by the STAT3 pathway and NF-*κ*B pathway can induce the expression of many inflammatory mediators, and this is a common inflammatory pathway that participates in the inflammatory response [23]. NF-*κ*B pathway activation promotes the secretion of inflammatory factors in the inflammatory response, which is essential in atherosclerosis [24, 25]. The STAT3 pathway is strongly related to atherosclerosis, and activation of the STAT3 pathway is critical for the effects of endothelial cell function, macrophage polarisation responses, inflammation, and immune responses [24, 26, 27]. CXCL16 plays roles in the inflammatory response, cell proliferation and migration, and angiogenesis by promoting STAT3 phosphorylation and activating the STAT3/NF-*κ*B pathway in islet-related inflammatory reactions (such as Type I diabetes) and tumour-related inflammatory reactions [28, 29]. The STAT3/NF-*κ*B pathway is involved in the inflammatory response associated with tumours and the inflammatory response associated with intestinal diseases. It also contributes to the reciprocal regulation of the release of the inflammatory factors IL-17A and TGF-*β* by macrophages [30–32].

**Figure 1:**
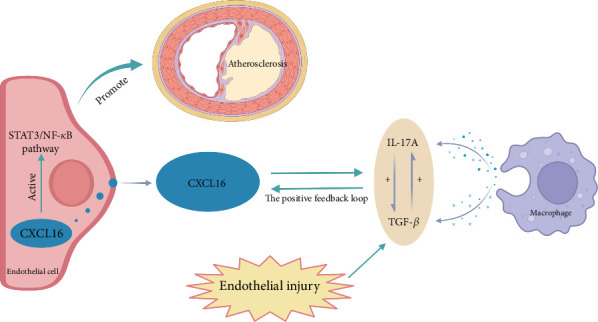
Molecular mechanism involved in carotid atherosclerosis.

To the best of the authors' knowledge, the specific mechanism by which CXCL16 promotes atherosclerosis is unknown. Our study explored the correlations among the inflammatory factors IL-17A and TGF-*β*, CXCL16, and the STAT3/NF-*κ*B pathway in atherosclerotic diseases in vivo and established an atherosclerotic cell model in which oxidized low-density lipoprotein (ox-LDL) was used to stimulate and elicit inflammatory factors, such as IL-17A and TGF-*β*, in Tohoku Hospital Pediatrics-1 (THP-1) cells [33]. This study further elucidates the mechanism by which the inflammatory factor CXCL16 and the STAT3/NF-*κ*B pathway participate in the inflammatory response in carotid atherosclerosis (Figure 1).

## 2. Materials and Methods

### 2.1. Ethics Statement

All the patients who participated in this study provided informed consent. This study was conducted in accordance with the principles of the Declaration of Helsinki and was approved by the Ethics Committee of the Affiliated Hospital of Qingdao University.

### 2.2. Collection and Analysis of Carotid Intima Media Samples From Patients With Atherosclerosis

From June 2021 to December 2021, we collected carotid intima tissues from 20 patients who underwent carotid endarterectomy. All patients who underwent carotid endarterectomy underwent brain CT and brain MRI, which included sequences of T1, T2, FLAIR (fluid-attenuated inversion recovery), diffusion-weighted imaging (DWI), apparent diffusion coefficient (ADC), magnetic resonance angiography (MRA), carotid artery ultrasound, cardiac ultrasonography, and brain and cervical computed tomography angiography (CTA). Carotid plaque stability was assessed according to carotid artery stenosis associated with ipsilateral carotid artery–related acute ischaemic stroke events via carotid ultrasound, and the patients were divided into a stable plaque group (*n* = 10) and a vulnerable plaque group (*n* = 10) [34]. There were no obvious differences between the two groups in terms of sex or age. The carotid endothelial tissue removed after carotid endarterectomy was frozen in liquid nitrogen for the shortest possible time, and paraffin-embedded sections were generated.

#### 2.2.1. Immunohistochemistry

Immunohistochemical analysis of CXCL16 was performed. After the carotid intimal tissue sections were paraffin-embedded, antigen repair was performed. Immunohistochemical staining was performed using an anti-CXCL16 antibody (1 mg/mL) (endothelial cell marker, rabbit polyclonal [Abcam]). After incubation with the corresponding secondary antibody (with an enzyme label), a freshly prepared DAB solution was used to develop the colour, and a haematoxylin dye solution was used to stain the nucleus. The slides were dehydrated and sealed. The secondary antibody used was enzyme-labelled rabbit anti-goat IgG (Servicebio). Image-Pro Plus 6.0 (Media Cybernetics, USA) software was used to analyse the surface density, positive rate, and histochemical score of CXCL16.

#### 2.2.2. Measurement of CXCL16 Expression in Carotid Artery Plaques by Western Blotting (WB)

Fifty milligrams of carotid intima tissue and 1000 *μ*L of lysis buffer (RIPA:PMSF = 100:1, Solarbio) were placed in a 50-mL centrifuge tube and completely homogenised on ice with a handheld grinder until a uniform lysate was obtained. The lysed sample was centrifuged at 12,000 r for 15 min, and the supernatant was added to SDS–PAGE sample loading buffer (2×) (ACE) at a ratio of 2:1. The supernatant was incubated at 95°C in a dry ThermoSTAT3 for 5 min and then frozen at −20°C. The extracted proteins were separated by FuturePAGE protein prefabricated gel electrophoresis and transferred to a polyvinylidene fluoride membrane (Millipore), which was blocked with milk (2.5 g of milk powder in 50 mL of double distilled water) at room temperature for 2 h. The membrane was incubated with a primary antibody (1:1000 dilution, CXCL16 antibody from Affinity) at 4°C for 8 h. Then, a secondary antibody from Proteintech was diluted at 1:2000, added to the membrane, and incubated at room temperature for 2 h, and the protein bands on the blot were visualized by white LED's bar Epi-IL lamination under the action of enhanced chemiluminescence (ECL) hypersensitivity reagent (Yeasen). The intensity of each band was measured by ImageJ software and standardised to the GAPDH expression level. The relative expression in the blank control was set to 1.

#### 2.2.3. Measurement of CXCL16 Messenger RNA (mRNA) Expression in Carotid Plaques by Reverse Transcription Polymerase Chain Reaction (RT–PCR)

Fifty milligrams of tissue was mixed with 1000 *μ*L of AG RNAex Pro RNA extraction reagent (Accurate Biotechnology (Hunan) Co., Ltd.) in a 50-mL centrifuge tube and thoroughly homogenised on ice with a handheld grinder until a uniform lysate was obtained. The lysed sample was placed in a centrifuge for 5 min and centrifuged at 12,000 r for 5 min. Total RNA was extracted by pipetting the supernatant. Then, the lysed sample was subjected to reverse transcription using an Accurate Biotechnology (Hunan) Co., Ltd. kit. The RNA was reverse transcribed into cDNA, and the obtained sample was subjected to quantitative PCR: initial denaturing at 95°C for 10 s, then amplifying with 40 cycles of 95°C for 5 s and 55°C for 30 s. The RT–PCR kit was purchased from Tsingke. RT–PCR was performed using a Roche LightCycler 480 II. The relative expression quantity of mRNAs was calculated by the 2^−ΔΔCt^ method, where ΔCt = Ct target gene − Ct internal reference gene and ΔΔCt = ΔCt experimental group − ΔCt control group (Table 1).

### 2.3. Cell Culture

Human umbilical vein endothelial cells (HUVECs) were used as model ECs. HUVECs were cultured in PUMCHUVEC T1 cell-specific medium (Procell, Wuhan, China). THP-1 cells were cultured in THP-1 cell culture medium (Procell, Wuhan, China). All the cells were cultured in a CO_2_ incubator (WCI-180, Wiggens) in a humid environment with 5% CO_2_ at 37°C. HUVECs were purchased from Wuhan Punosi Life Science and Technology Co., Ltd. (cell name: Pum-HuVEC-T1; cell batch: CL-0675; cell number: 1 × 10^6^). THP-1 cells were purchased from Wuhan Punosi Life Science and Technology Co., Ltd. (cell name: THP-1; cell batch: CL-0233; cell number: 1 × 10^6^).

### 2.4. Oil Red O Staining

After the transformation of THP-1 cells into macrophages via stimulation with phorbol 12-carnitate 13-acetate (PMA) (MedChemExpress, USA), ox-LDL (Yiyuan Biotechnologies, Guangzhou, China) was added to induce their transformation into foam cells, and the transformation was verified by staining with Oil Red O. The cells were incubated with Oil Red O staining solution and Mayer's haematoxylin staining solution, and lipid droplets were observed under an inverted microscope. The Oil Red O Dye Solution Kit was obtained from Solarbio.

### 2.5. Transfection

The cells were inoculated into 24-well plates using liposomal nucleic acid transfection reagent (Shanghai Yeison Biotechnology Co., Ltd.) according to the manufacturer's guidelines. During plasmid transfection, 0.5 *μ*g of plasmid and 2 *μ*L of transfection medium were added to each well. During small interfering RNA (siRNA) transfection, 20 pmol of siRNA and 1 *μ*L of transfection reagent were added to each well, and the cells were cultured in serum-free medium for 4 h; then, the medium was replaced with complete medium for further culture. Fluorescence was observed after 3 days under a 10× magnifying glass. IL-17A, TGF-*β*, and CXCL16 plasmids were all purchased from Gekkai Gene, and IL-17A, TGF-*β*, and CXCL16 siRNAs were all purchased from RiboBio.

### 2.6. Coculture System

Transwell chambers (0.4 *μ*m) were purchased from JET BIOFIL (China). To study inflammatory factors, macrophages were inoculated in the lower compartment, and HUVECs were inoculated in the upper compartment. The addition of 25 *μ*g/mL ox-LDL to the lower chamber created an inflammatory environment. After 24 h of cell coculture, the total protein was extracted for analysis. To study the CXCL16 and STAT3/NF-*κ*B pathways, HUVECs were inoculated in the lower compartment, and macrophages were inoculated in the upper compartment. The addition of 25 *μ*g/mL ox-LDL to the lower chamber created an inflammatory environment. Protein and mRNA were extracted and analysed after 24 h of cell coculture.

### 2.7. The Expression Levels of IL-17A and TGF-*β* by Macrophages Determined by Enzyme-Linked Immunosorbent Assay (ELISA)

We measured the expression levels of IL-17A and TGF-*β* by macrophages using a 96-well human interleukin-17A ELISA kit (Elabscience, Wuhan, China) and a 96-well transforming growth factor-*β*1 ELISA kit (Elabscience, Wuhan, China) [32] following the manufacturer's guidelines. Before the start of the experiment, an activation reagent was used to activate the TGF-*β* in the sample.

### 2.8. The Expression of CXCL16 and Factors Related to the STAT3/NF-*κ*B Pathway Was Determined by WB

Proteins were extracted from HUVECs, separated by FuturePAGE protein gel electrophoresis using precast gels, and then transferred to polyvinylidene difluoride membranes (Millipore, USA). The membrane was blocked with milk (2.5 g milk powder in 50 mL double distilled water) at room temperature for 2 h. The cells were incubated with a primary antibody (diluted 1:1000, anti-CXCL16 antibody from Affinity) at 4°C for 8 h. Then, the membranes were incubated with secondary antibodies from Proteintech that were diluted at 1:2000 at room temperature for 2 h. Then, the protein bands on the membranes were visualized with white LED's bar Epi-IL fluorescence under the action of ECL hypersensitive chromogenic reagent (Yeasen, Shanghai, China). The intensities of the bands were measured using ImageJ software. The expression of CXCL16 and NF-*κ*B was standardised to that of GAPDH. The expression of STAT3 and phospho-STAT3 (p-STAT3) was standardised to that of actin. The relative expression in the blank control was set to 1.

### 2.9. CXCL16 mRNA Expression in HUVECs Was Measured by RT–PCR

Total RNA was extracted from HUVECs and then reverse transcribed into cDNA by a kit from Accurate Biotechnology (Hunan) Co., Ltd. The resulting sample was used for quantitative PCR: initial denaturation at 95°C for 10 s, then 40 cycles of 95°C for 5 s, and 55°C for 30 s. The RT–PCR kit was purchased from Tsingke. RT–PCR was performed using a Roche LightCycler 480 II. The relative expression quantity of mRNAs was calculated by the 2^−ΔΔCt^ method, where ΔCt = Ct target gene −− Ct internal reference gene and ΔΔCt = ΔCt experimental group −− ΔCt control group.

### 2.10. Cell Counting Kit-8

The proliferative ability of the cells was detected by a CCK-8 assay (MedChemExpress, USA). After transfection, the cells were seeded in 96-well plates (2 × 10^4^ cells/well). In accordance with the instructions of the CCK-8 assay, CCK-8 solution was added at 0, 24, 48, 72, or 96 h, and the plates were cultured in the cell incubator for 4 h. The absorbance was measured at 450 nm according to the manufacturer's instructions.

### 2.11. Wound-Healing Assay

HUVECs were inoculated in Transwell compartments and cultured in 90% DMEM (containing 10% foetal bovine serum and 1% penicillin–streptomycin). When the density of the HUVECs reached 90%, after the medium was removed from the lower chamber, a 200-*μ*L sterile pipetting gun was used to create a wound in the HUVECs in the lower chamber. The dissociated cells were washed away with PBS, and then, 500 *μ*L of 90% DMEM (with 10% foetal bovine serum and 1% penicillin–streptomycin) was added again. Macrophages were seeded in the upper chamber of the Transwell plate and cocultured with endothelial cells. Wound healing was observed 12 h later. The results were analysed using ImageJ software. Relative healing area = (initial area − final area)/initial area. The data are shown as the means ± standard deviations.

### 2.12. Statistical Analysis

All the experiments were performed in triplicate and analysed with GraphPad Prism Version 9.3.0 (345) (GraphPad Software, Boston, MA, USA). All the data are expressed as the means ± SDs. The *t*-tests and ANOVA were used to analyse the differences between groups. *p* < 0.05 was considered a statistically significant difference.

## 3. Results

### 3.1. Carotid Intima

**Figure 2:**
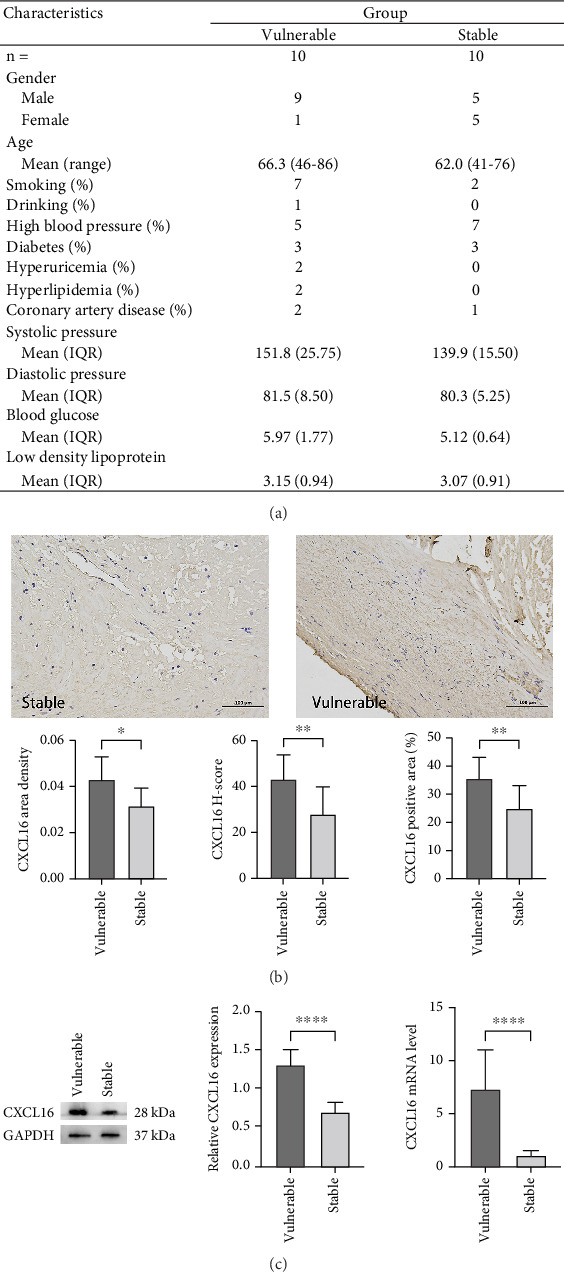
Assessment of CXCL16 expression in plaques by immunohistochemical staining. (a) Patient general characteristics. (b) Immunohistochemical analysis. Immunohistochemical staining of carotid plaque intima was performed using a CXCL16 antibody, and surface density (accumulated optical density value (IOD)/tissue area to be measured), histochemical score (H-score), and positive rate (number of positive cells/total number of cells) were analysed. The unpaired *t*-test was used to analyse the differences between the two groups, with all the data presented as the means ± SDs. *n* = 10; ⁣^∗^*p* < 0.05; ⁣^∗∗^*p* < 0.01. (c) The expression levels of CXCL16 in vulnerable plaques and stable plaques were compared by WB and RT–PCR. An unpaired *t*-test was used to analyse the differences between the two groups, with all data presented as the means ± SDs. *n* = 10, ⁣^∗∗∗∗^*p* < 0.0001.

We aimed to verify whether the expression level of CXCL16 differed between stable and vulnerable plaques. We first performed immunohistochemical staining of plaque tissues from the stable and vulnerable plaque groups (Figure 2). The surface density, positive rate, and histochemical score (H-core) of CXCL16 in the vulnerable plaque group and the stable plaque group were analysed. Then, we extracted protein and mRNA by grinding the plaque tissue. WB and RT–PCR were used to detect CXCL16 expression levels (Figure 1). The results revealed that the expression level of CXCL16 in vulnerable plaques was significantly greater than that in stable plaques. These results suggest that CXCL16 may be a risk factor for carotid atherosclerotic plaque stability.

### 3.2. Establishment of the Atherosclerosis Cell Model

**Figure 3:**
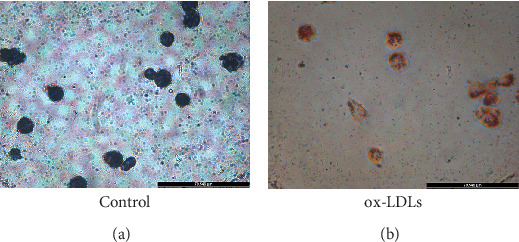
Oil Red O staining. (a) Blank control group. (b) Ox-LDL treatment group.

To establish a model of atherosclerotic cells, we selected THP-1 cells. PMA is a PKC agonist, and THP-1 cells can be induced to differentiate into macrophages under the action of PMA. The accumulation of fat in macrophages can lead to the transformation of macrophages into high-volume foam cells. We induced the transformation of macrophages into foam cells by adding ox-LDL and stained them with Oil Red O. Microscopically, cells in the ox-LDL-stimulated group presented significant lipid droplets compared with those in the control group, demonstrating successful foam cell transformation (Figure 3).

### 3.3. Synergism of Inflammatory Factors Secreted by Macrophages

**Figure 4:**
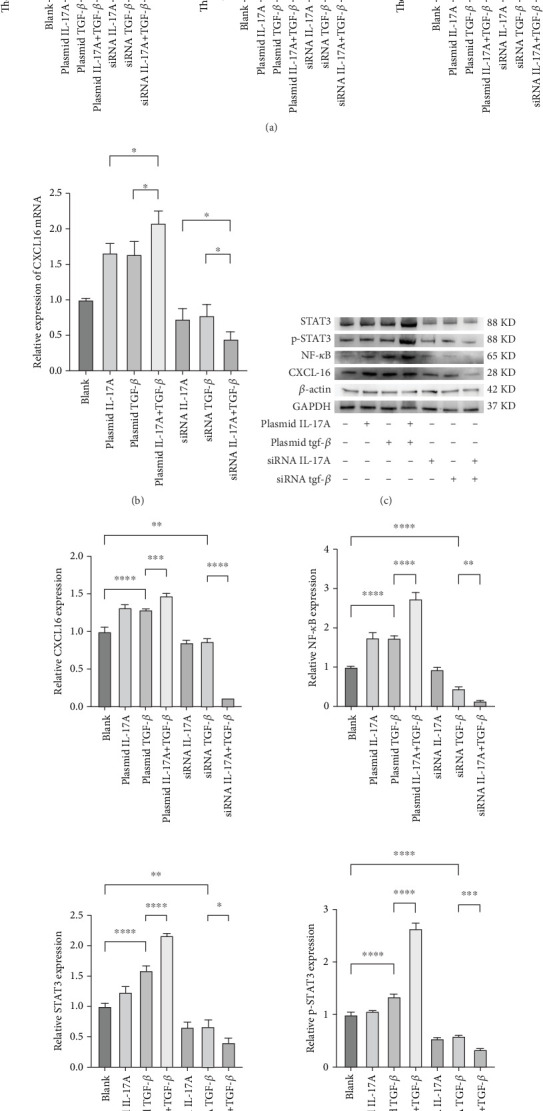
Macrophages cocultured with HUVECs after regulating the expression levels of IL-17A and TGF-*β*. (a) The expression levels of CXCL16, IL-17A, and TGF-*β* were analysed by ELISA. One-way ANOVA was used to analyse the differences between the groups, and the data are shown as means ± SDs. *n* = 6; ⁣^∗^*p* < 0.05; ⁣^∗∗^*p* < 0.01; ⁣^∗∗∗^*p* < 0.001; ⁣^∗∗∗∗^*p* < 0.0001. (b) The expression of CXCL16 mRNA was detected by RT–PCR. One-way ANOVA was used to analyse the differences between the groups, and the data are shown as means ± SDs. *n* = 3; ⁣^∗^*p* < 0.05. (c, d) The expression levels of CXCL16, STAT3, p-STAT3, and NF-*κ*B were measured by WB. One-way ANOVA was used to analyse the differences between the groups, and the data are shown as means ± SDs. *n* = 3; ⁣^∗^*p* < 0.05; ⁣^∗∗^*p* < 0.01; ⁣^∗∗∗^*p* < 0.001; ⁣^∗∗∗∗^*p* < 0.0001.

First, to determine the relationships among the inflammatory cytokines secreted by macrophages, we modulated the expression of the inflammatory cytokines IL-17A and TGF-*β* by transfection. After successful transfection, the cells were cocultured with HUVECs. The expression levels of IL-17A and TGF-*β* were analysed by ELISA (Figure 4a). ELISAs revealed that TGF-*β* was highly expressed when IL-17A was highly expressed, and the expression of TGF-*β* was reduced when I-17A was silenced. Similarly, the expression of IL-17A increased with high expression of TGF-*β*, whereas the expression of IL-17A decreased with silencing of TGF-*β*. Therefore, the inflammatory factors IL-17A and TGF-*β* can mutually affect each other's expression.

### 3.4. The Synergistic Effect of Macrophage-Derived Inflammatory Factors on the Expression of CXCL16 and STAT3/NF-*κ*B Pathway Components in Endothelial Cells Has an Amplification Effect

Next, to test whether macrophage-secreted inflammatory factors increase the expression of CXCL16, STAT3, p-STAT3, and NF-*κ*B, we cocultured HUVECs with macrophages whose expression levels of IL-17A and TGF-*β* were deliberately altered. First, the level of CXCL16 in the coculture medium was detected by ELISA (Figure 4a). The results revealed that increases in IL-17A and TGF-*β* expression promoted the expression of CXCL16 in HUVECs and that decreases in IL-17A and TGF-*β* expression inhibited the expression of CXCL16 in HUVECs.

The expression levels of CXCL16, NF-*κ*B, STAT3, and p-STAT3 in HUVECs were detected by RT–PCR and WB (Figure 4b, 4c, and 4d). The results revealed that increased expression levels of IL-17A and TGF-*β* promoted the expression of CXCL16, NF-*κ*B, STAT3, and p-STAT3 in HUVECs and that the promoting effect was more obvious when the expression levels of IL-17A and TGF-*β* were increased simultaneously. However, decreased expression levels of IL-17A and TGF-*β* inhibited the expression of CXCL16, NF-*κ*B, STAT3, and p-STAT3 in HUVECs, and the inhibitory effect was more obvious when the expression levels decreased simultaneously. These results suggest that IL-17A and TGF-*β* not only positively regulate the expression of CXCL16 and the STAT3/NF-*κ*B pathway in endothelial cells under inflammatory conditions but also have a synergistic effect on the inflammatory cytokines IL-17A and TGF-*β*. The expression of CXCL16 and STAT3/NF-*κ*B pathway–related factors can be further amplified.

### 3.5. Macrophage-Derived Inflammatory Factors Positively Regulate CXCL16 and Activate the STAT3/NF-*κ*B Pathway

**Figure 5:**
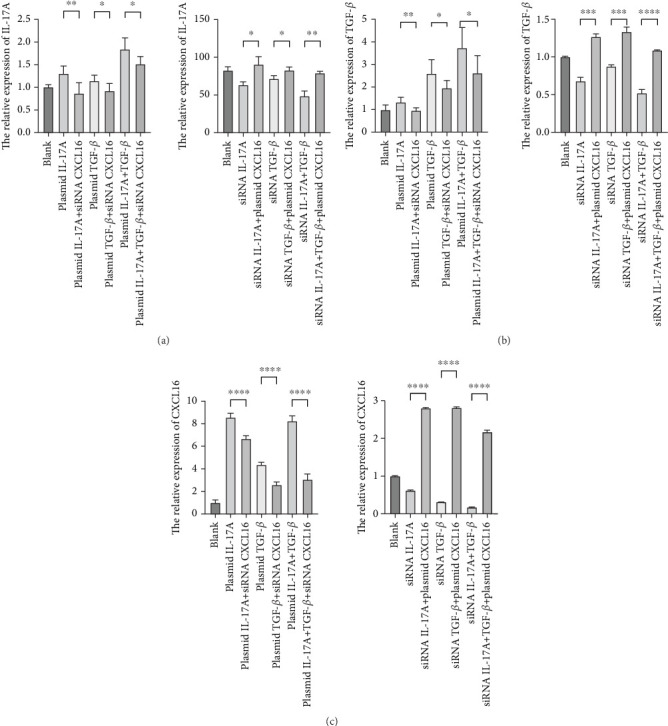
HUVECs with controlled expression of CXCL16 were cocultured with macrophages with controlled expression of IL-17A and TGF-*β*. (a–c) The expression levels of CXCL16, IL-17A, and TGF-*β* were determined by ELISA. One-way ANOVA was used to analyse the differences between the groups, and the data are shown as means ± SDs. Group A, *n* = 7.

To verify the positive feedback regulatory loop between the inflammatory factors IL-17A and TGF-*β* and CXCL16, we changed the expression level of CXCL16 in HUVECs by altering the expression levels of IL-17A and TGF-*β* in THP-1 cells and placed the two in a coculture system. The levels of CXCL16, IL-17A, and TGF-*β* released into the coculture system were detected by ELISA (Figure 5a, 5b, and 5c). The results revealed that the expression levels of IL-17A and TGF-*β* in macrophages increased after the upregulation of CXCL16 and decreased after the downregulation of CXCL16. Therefore, the above results suggest that the inflammatory cytokines IL-17A and TGF-*β* can promote the expression and release of CXCL16 into the inflammatory environment. When the CXCL16 content is increased in the inflammatory environment, the signal of increased CXCL16 expression can be fed back to inflammatory cells to further promote the expression and release of IL-17A and TGF-*β*. The interactions among the three form a positive feedback regulation cycle.

**Figure 6:**
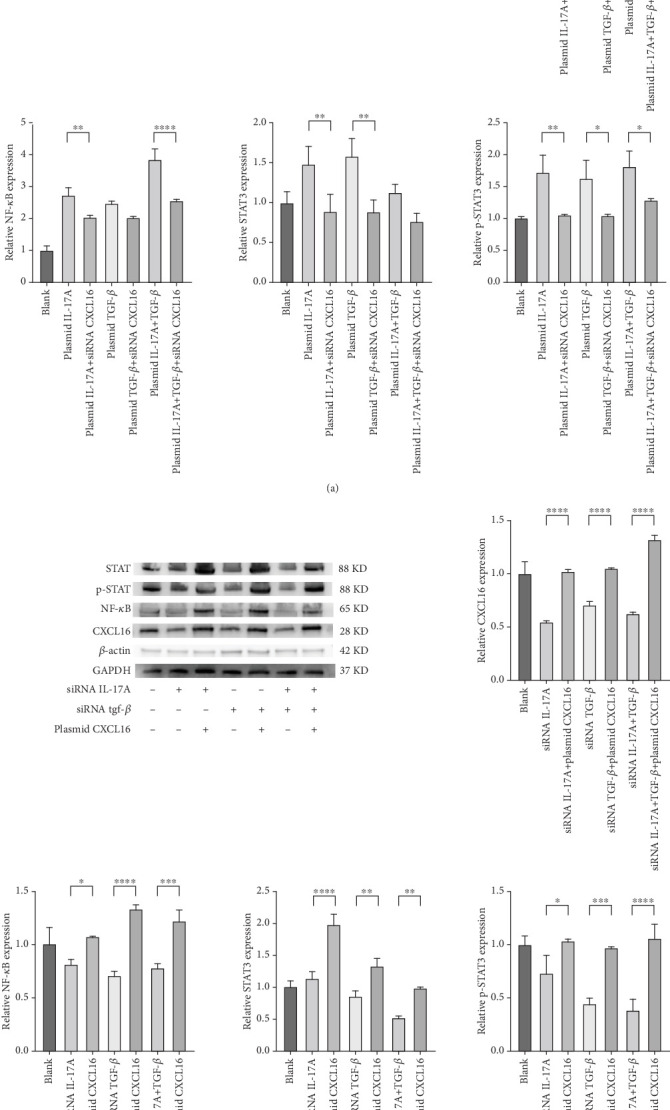
HUVECs with controlled expression of CXCL16 were cocultured with macrophages with controlled expression of IL-17A and TGF-*β*. (a, b) The expression levels of CXCL16-, NF-*κ*B-, STAT3-, and p-STAT3-related factors were measured by WB, and the differences among the groups were analysed by one-way ANOVA. The data are shown as means ± SDs, *n* = 3.

The expression levels of CXCL16, NF-*κ*B, STAT3, and p-STAT3 were detected by WB (Figure 6a,b). The results revealed that the expression levels of NF-*κ*B, STAT3, and p-STAT3 increased with increasing IL-17A and TGF-*β* and decreased with decreasing CXCL16. Similarly, the expression levels of NF-*κ*B, STAT3, and p-STAT3 decreased with decreasing IL-17A and TGF-*β*, and the expression levels of NF-*κ*B, STAT3, and p-STAT3 increased with increasing CXCL16. These results indicate that CXCL16 has a positive regulatory effect on STAT3 and NF-*κ*B pathway-related factors and that CXCL16 has a stronger regulatory effect on STAT3 and NF-*κ*B pathway-related factors than on IL-17A and TGF-*β*. After adjusting the expression level of CXCL16, the expression levels of the STAT3 pathway-related and NF-*κ*B pathway-related factors changed more significantly. These results suggest that there is a synergistic effect between the inflammatory factors IL-17A and TGF-*β* in the inflammatory environment, which amplifies the increase in CXCL16 expression. CXCL16 can promote the expression of the inflammatory cytokines IL-17A and TGF-*β* through a positive feedback regulatory loop, and the three can promote the expression of each other through this positive feedback regulatory loop, leading to further activation of the STAT3 pathway and NF-*κ*B pathway to promote the expression of STAT3 pathway-related and NF-*κ*B pathway-related factors.

### 3.6. Wound-Healing Assay

**Figure 7:**
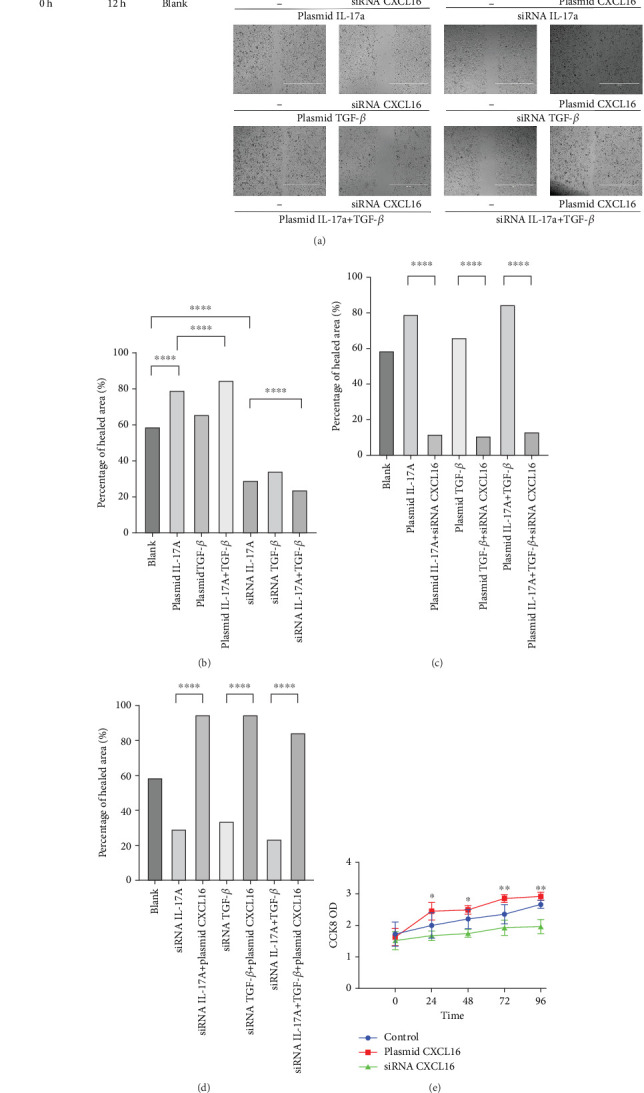
Wound-healing assay and CCK-8 assay. (a) HUVEC migration was evaluated by a wound-healing assay. (b–d) Quantification of the wound-healing assay results. One-way ANOVA was used to analyse the differences between groups, and the data are shown as means ± SDs. *n* = 6. (e) HUVEC proliferation was evaluated by a CCK-8 assay, *n* = 3.

The migration of HUVECs was observed with wound-healing experiments [35, 36](Figure 7a, 7b, 7c, and 7d). Compared with the 58.9% ± 1.05% of the control cells, the cells upregulated by IL-17A accounted for 79.14% ± 0.96% of the closed wound area (*p* < 0.0001), the cells downregulated by IL-17A accounted for 29.41% ± 1.14% of the closed wound area (*p* < 0.0001), and the cells downregulated by IL-17A accounted for 29.41% ± 1.14% of the closed wound area. The percentage of cells with upregulated CXCL16 expression caused by downregulated IL-17A was close to 94.77% ± 0.88% of the closed wound area (*p* < 0.0001); the percentage of cells with upregulated TGF was close to 65.87% ± 1.79% of the closed wound area (*p* < 0.0001). The TGF-treated cells accounted for 33.96% ± 1.21% of the closed wound area (*p* < 0.0001), whereas the cells whose CXCL16 expression was upregulated in response to TGF-*β* were downregulated in nearly 94.65% ± 0.99% of the closed wound area (*p* < 0.0001), which proved that macrophage-derived inflammatory cytokines and CXCL16 expression can promote HUVEC migration.

### 3.7. Cell Proliferation

As depicted in the figure, the CCK-8 optical density (OD) 450 values of the endothelial cells that were transfected with the CXCL16 plasmid, those that were transfected with siRNA, and the control cells were measured at each test point during the test time (24–72 h); the OD value of the plasmid-transfected group was obviously greater than that of the control group, whereas the OD value of the siRNA-transfected group was obviously lower than that of the control group (Figure 7e). These results suggest that upregulation of CXCL16 expression in endothelial cells can promote their proliferation, whereas inhibition of CXCL16 expression can inhibit their proliferation. Therefore, the regulation of CXCL16 expression can significantly affect the proliferation of endothelial cells.

## 4. Discussion

This study investigated the involvement of CXCL16 in the development of carotid atherosclerosis and the related molecular mechanisms. These findings indicate that CXCL16 is a key factor in promoting carotid atherosclerosis and plaque instability. Moreover, a positive feedback loop exists between CXCL16 and inflammatory factors such as IL-17A and TGF-*β*, mutually promoting their expression and accelerating carotid atherosclerosis progression via activation of the STAT3 and NF-*κ*B pathways.

CXCL16 is a common chemokine and adhesion factor in the inflammatory response. Andersen et al. analysed the relationship between CXCL16 levels and cardiovascular mortality and spontaneous myocardial infarction through PLATO experiments. A previous study revealed that CXCL16, which acts as an independent risk factor, significantly elevates the likelihood of cardiovascular diseases [10]. Shibata et al. induced atherosclerosis by feeding Apo-E knockout mice a high-fat diet; studied early atherosclerotic lesions, progressive lesions, and diseased plaques; and reported that CXCL16 was not expressed in normal arteries but gradually increased in different atherosclerotic lesions [37]. Therefore, CXCL16 participates in the pathological progression of atherosclerosis. By establishing a mouse model of CXCL16 overexpression, Zhao, Yang, and Wu further demonstrated that CXCL16 promotes atherosclerosis [38].

However, the present studies only support the idea that CXCL16 contributes to the development of carotid atherosclerosis in humans. To date, no studies have directly demonstrated that CXCL16 promotes the development of carotid atherosclerosis and affects its stability. The present study directly analysed carotid plaques via immunohistochemical staining, WB, and RT–PCR and compared the differences in CXCL16 expression levels between stable and unstable plaques, revealing significantly greater expression of CXCL16 in unstable plaques than in stable plaques. These results demonstrated that CXCL16 is an influential factor that promotes carotid atherosclerosis and plaque instability in vivo.

Previously, domestic and foreign studies generally reported that the formation of carotid atherosclerotic plaques occurs in an inflammatory state. A high lipid state and other risk factors lead to endothelial damage, increased permeability, and an inflammatory response. Prolonged inflammatory stimulation results in increased numbers of macrophages and lymphocytes, the uptake of ox-LDL by macrophages to form foam cells, and the migration and proliferation of endothelial cells. This leads to the continuous progression of carotid atherosclerosis and eventually plaque rupture [39–45]. Accumulating evidence indicates that a wide range of inflammatory factors play crucial roles in the development of carotid atherosclerosis. For example, IL-17A and TGF-*β*, two important inflammatory factors in the inflammatory response, are expressed mainly in endothelial cells, smooth muscle cells, macrophages, and T cells in carotid atherosclerotic plaques and are involved in atherosis-related inflammatory reactions [16, 46]. CXCL16 promotes the release of the inflammatory factor IL-17A in the inflammatory response after myocardial ischaemia–reperfusion injury [21]. In the autoimmune inflammatory response of the central nervous system, the inflammatory factor IL-17A also promotes the expression of CXCL16 [47]. The present study revealed that the overexpression of IL-17A and TGF-*β* can promote the production and secretion of CXCL16 in HUVECs and that CXCL16 serves as a proliferative signal and regulatory inducer, promoting both the proliferation and migration of endothelial cells. On the other hand, the overexpression of CXCL16 also promoted the release of IL-17A and TGF-*β*. Our findings indicate that CXCL16, IL-17A, and TGF-*β* levels can be correlated with each other, forming a positive feedback regulatory loop among the three factors and promoting the development of carotid atherosclerosis.

The NF-*κ*B pathway directly targets inflammation by increasing the production of inflammatory cytokines, chemokines, and adhesion molecules. Additionally, it regulates cell proliferation, differentiation, apoptosis, and morphogenesis [24, 48]. Notably, NF-*κ*B activation is essential for regulating genes involved in the inflammatory response of cells, which are crucial in atherosclerosis [25]. The STAT3 pathway plays a critical role in regulating leukocyte recruitment, foam cell formation, and the proliferation and migration of vascular smooth muscle cells [49, 50]. STAT3 serves as a significant transcription factor in both the immune response and inflammation [23, 51]. When STAT3 is phosphorylated by tyrosine, the generation of p-STAT3 regulates the transcription of many target genes [24, 49, 52]. These findings indicate that inflammatory factors and CXCL16 have regulatory effects on the expression of STAT3 and NF-*κ*B pathway-related cytokines. In summary, the related molecular mechanism by which CXCL16 promotes carotid atherosclerosis may involve CXCL16 and inflammatory factors promoting the expression of each other through a positive feedback regulatory loop and activating the pathway to further promote inflammation and endothelial cell proliferation and migration and promote the development of carotid atherosclerosis (Figure 1).

Our study links the pathogenesis of carotid atherosclerosis from the infiltration of macrophages, the expression and release of inflammatory factors and chemokines, and the activation of related inflammatory pathways to the proliferation and migration of endothelial cells and the further development of the inflammatory response, which has not been achieved in previous studies. Our study analysed CXCL16 and its mechanisms that contribute to pathophysiological changes during carotid atherosclerosis and plaque instability. This study contributes to the research and development of targeted drugs and provides new therapeutic approaches and strategies for the prevention and treatment of carotid atherosclerotic plaques and acute thromboembolism. Our study also has several limitations, including the relatively small number of participants with carotid plaques, so we need to recruit a larger sample size to confirm our conclusions. Second, our research on the molecular mechanism related to the promotion of carotid atherosclerosis by CXCL16 is limited to cell experiments and lacks verification in animal models. Therefore, our experiment is preliminary, and further research is still needed.

## Figures and Tables

**Table 1:** Primers used in this study.

**Genes**	**5**⁣′** -3**⁣′	**Primer sequences**
CXCL16	Forward	GCAGCGTCACTGGAAGTTGTTATTG
	Reverse	CCGATGGTAAGCTCTCAGGTGTTTC
ACTB	Forward	GCGGACTATGACTTAGTTGCGTTACA
	Reverse	TGCTGTCACCTTCACCGTTCCA

## Data Availability

The data generated in the present study may be requested from the corresponding authors.
